# Supplementary material for: Conditional chemoconnectomics (cCCTomics) as a strategy for efficient and conditional targeting of chemical transmission
Source: eLife. 2024 Apr 30;12:RP91927. doi: 10.7554/eLife.91927 (PMC11060718; doi:10.7554/eLife.91927)
Supplement: Figure 3—source data 3. [file elife-91927-fig3-data3.zip › Figure 3 source data3 part 2.zip!/Figure 3-Source data 3-part2/1053f3-1-CRISPResso_Report_t6djfp6u.zip!/CRISPResso_on_1053f3_1/9.Alleles_frequency_table_around_sgRNA_ggccaccagatgcaaaatcg.pdf]

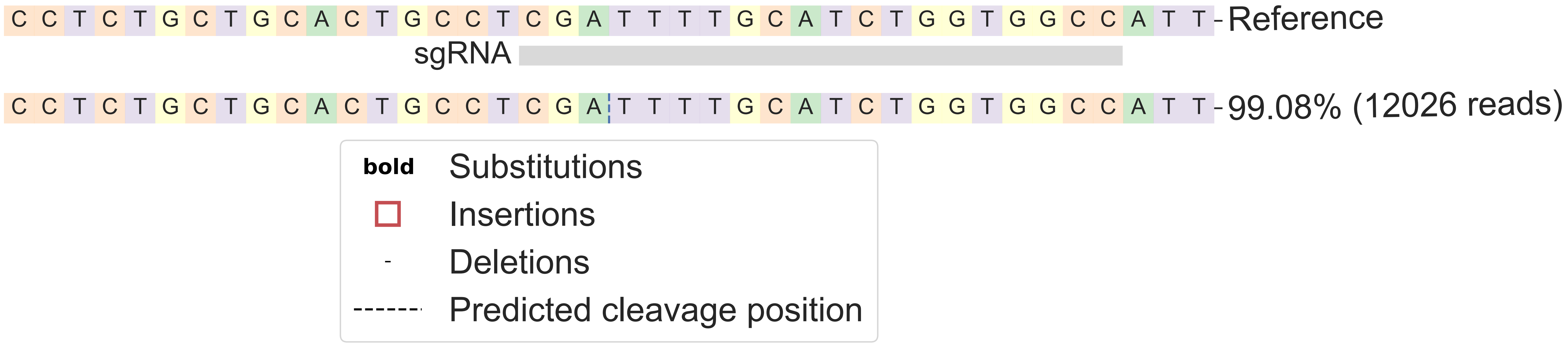
C C T C T G C T G C A C T G C C T C G A T T T T G C A T C T G G T G G C C A T T
Reference
sgRNA
C C T C T G C T G C A C T G C C T C G A T T T T G C A T C T G G T G G C C A T T
99.08% (12026 reads)
bold Substitutions
Insertions
Deletions
Predicted cleavage position
